# Exercise Cardiovascular Magnetic Resonance for the Diagnosis and Phenotyping of Heart Failure with Preserved Ejection Fraction: A Comprehensive Review

**DOI:** 10.3390/diagnostics16213462

**Published:** 2026-10-23

**Authors:** Waner Li, Caiyun Han, Jiahao Wu, Qi Wang, Hong Ren, Wei Deng

**Affiliations:** Department of Radiology, Sir Run Run Shaw Hospital, Zhejiang University School of Medicine, Hangzhou 310016, China; lyxer99@163.com (W.L.); hcy210505@163.com (C.H.); jiahaowu0110@163.com (J.W.); y223180029@zju.edu.cn (Q.W.)

**Keywords:** heart failure with preserved ejection fraction, exercise cardiovascular magnetic resonance, exercise haemodynamics, left atrial function, cardiovascular reserve, phenotyping

## Abstract

In heart failure with preserved ejection fraction (HFpEF), functional abnormalities may emerge mainly during exercise. Resting investigations may therefore explain exertional symptoms only partly. Exercise cardiovascular magnetic resonance (Ex-CMR) measures chamber volumes, deformation and blood flow during physiological stress to assess cardiovascular reserve. This review examines the technical foundations of Ex-CMR and its applications in HFpEF diagnosis, physiological phenotyping and treatment monitoring. It considers the physiological interpretation and clinical evidence for atrial, biventricular and vascular reserve indices, with attention to standardised acquisition, multiparametric assessment and clinical translation.

## 1. Introduction

HFpEF accounts for approximately half of heart failure cases, and its relative prevalence has increased in some population studies. This trend accompanies population ageing and the growing burden of obesity, hypertension, diabetes, atrial fibrillation and chronic kidney disease [1,2]. HFpEF involves more than diastolic dysfunction: abnormalities may affect cardiac structure, haemodynamics, the pulmonary circulation, vascular function and peripheral metabolic reserve. Patients may have substantial exertional symptoms despite preserved resting left ventricular ejection fraction. Left atrial volume, natriuretic peptide levels or echocardiographic filling-pressure surrogates may be borderline [3]. When resting assessment is inconclusive, changes in left-sided filling pressure, forward flow and pulmonary vascular load during exercise can help identify the physiological abnormalities underlying symptoms [4,5,6].

The H_2_FPEF and HFA-PEFF scores help estimate pre-test probability and identify patients requiring further investigation, but neither fully characterises exercise haemodynamics [4,5]. Exercise right heart catheterisation directly measures pulmonary capillary wedge pressure (PCWP), pulmonary artery pressure and cardiac output. In the appropriate clinical setting, these measurements provide a reference for diagnosing exercise-induced HFpEF. Resting PCWP ≥ 15 mmHg or supine exercise PCWP ≥ 25 mmHg commonly supports elevated left-sided filling pressure, interpreted in relation to posture, workload, respiratory phase, calibration and cardiac output response [6]. Its invasive nature limits routine first-line use in the large population presenting with exertional symptoms.

Stress echocardiography evaluates mitral inflow, tissue Doppler velocities, tricuspid regurgitation velocity and cardiac output during exercise or early recovery. Acoustic windows, arrhythmias and the interval between exercise cessation and imaging can affect its results [5,7]. Cardiopulmonary exercise testing (CPET) objectively measures exercise limitation, although it often cannot distinguish the contributions of the left heart, pulmonary circulation and peripheral tissues. Resting CMR provides reproducible measurements of ventricular and atrial volumes, late gadolinium enhancement (LGE), native T1 and extracellular volume (ECV), and assesses pericardial or infiltrative disease. However, conventional resting breath-hold cine imaging may also miss impaired functional reserve [8].

Ex-CMR adds to resting structural assessment by measuring how atrial function, biventricular volumes and output, and great-vessel blood flow change under stress. The 2021 HFpEF-Stress Trial compared real-time exercise CMR with exercise right heart catheterisation. Exercise left atrial long-axis strain (LA-LAS) showed high diagnostic discrimination [9]. Subsequent medium-term and four-year follow-up reports examined the association between atrial function and clinical events [10,11]. This review examines how Ex-CMR measurements inform diagnosis and physiological phenotyping, and how the evidence may translate into clinical practice.

## 2. Literature Search and Evidence Appraisal

A structured literature search was completed on 1 September 2026 in MEDLINE, Embase, Web of Science Core Collection, and Scopus, covering records from database inception to that date. The strategy combined controlled vocabulary and free-text terms for heart failure with preserved ejection fraction (HFpEF), cardiovascular magnetic resonance (CMR), and exercise or exercise stress. A complementary technical search included terms related to real-time imaging, feasibility, reproducibility, reference values, phase-contrast flow imaging, feature tracking, compressed-sensing and deep-learning reconstruction, spectroscopy, phosphorus magnetic resonance spectroscopy, blood oxygen level-dependent imaging, and oxygen measurements. Database-specific search strategies are provided in Supplementary Table S1. Reference lists of eligible articles and relevant reviews were screened, and forward citation searching of key Ex-CMR reports was undertaken to identify additional studies.

Eligible reports were full-text English-language human studies that evaluated in-magnet exercise CMR or CMR acquired immediately after dynamic exercise in patients with suspected or established HFpEF. Technical studies in healthy participants or comparator populations were included when they directly informed Ex-CMR acquisition, reproducibility, feasibility, or interpretation of exercise-related parameters. We excluded animal and paediatric studies, case reports, editorials, narrative reviews, conference abstracts without a corresponding full report, pharmacological stress studies without an exercise component, and studies without a relevant CMR-based exercise assessment. Studies using resting CMR alone were considered only when they informed differential diagnosis, technical interpretation, or the physiological context of exercise findings.

For each included study, we recorded the study design, sample size, participant characteristics, HFpEF definition or comparator group, exercise protocol, CMR acquisition timing, reference standard or clinical endpoint, analyzable scan rate, exclusions, principal quantitative findings, and follow-up outcomes where available. Because the literature spans diagnostic validation, physiological phenotyping, technical feasibility, prediction modelling, prognosis, and small mechanistic intervention studies, formal pooling was not appropriate.

Evidence was appraised using a structured narrative framework rather than a single risk-of-bias instrument. Studies were interpreted according to the appropriateness of their reference standard, cohort size, selection of participants, timing of CMR relative to exercise and invasive testing, completeness of analyzable data, independence of validation, and relevance to clinical decision-making. We distinguished direct diagnostic evidence using invasive exercise haemodynamics, exploratory imaging or prediction-model studies, technical and reference-range studies, and prognostic or intervention studies. Reports originating from the HFpEF-Stress research programme were considered related analyses of overlapping or partially overlapping participants rather than independent replications. Table 1 (Section 5.5) provides an overview of the design, reference standard, CMR acquisition stage, and cohort relationships of the principal Ex-CMR studies. A fuller study-level appraisal is provided in Supplementary Table S2.

## 3. Pathophysiology of HFpEF and Mechanisms of Exercise Intolerance

### 3.1. Cardiac Dysfunction and Impaired Cardiovascular Coupling

Several cardiac mechanisms may contribute to exercise intolerance in HFpEF, including a steeper diastolic pressure–volume relationship, reduced left ventricular compliance and a shorter diastolic filling period. During exercise, the healthy heart maintains forward output through increased venous return, diastolic suction, heart rate, and contractile reserve. In HFpEF, limited diastolic filling time may restrict end-diastolic volume recruitment. Left atrial pressure may then rise without an effective increase in stroke volume as workload increases [12,13]. The left atrium reflects the chronic filling-pressure burden and transfers pulmonary venous return to the left ventricle during exercise. Impaired atrial reservoir function and atrioventricular coupling may promote an earlier rise in pressure during exercise [9,11,14,15,16]. Preserved left ventricular ejection fraction does not imply normal contractile reserve. Exercise responses in longitudinal, circumferential or torsional mechanics, and derived measures such as haemodynamic forces, may reveal limitations concealed by resting ejection fraction [17,18]. The right ventricle must accommodate increased pulmonary blood flow and changes in pulmonary vascular resistance and compliance during exercise. Limited right ventricular contractile reserve, higher pulmonary arterial afterload, and impaired right ventricular–pulmonary arterial coupling may jointly reduce cardiac output [19,20,21,22,23]. Systemic arterial stiffness and abnormal ventricular–arterial coupling may further increase the load against which the left ventricle ejects during exercise [24,25].

### 3.2. Extracardiac Contributors and Syndrome Heterogeneity

Left ventricular diastolic function alone cannot explain the symptoms of HFpEF. Pulmonary vascular or parenchymal disease, anaemia, obesity, deconditioning, impaired skeletal muscle oxygen utilisation and endothelial dysfunction can cause or exacerbate exertional dyspnoea. Obesity and epicardial adipose tissue may affect cardiac structure and function through inflammation, mechanical restraint and metabolic alterations [26]. Phosphorus magnetic resonance spectroscopy has identified myocardial energetic abnormalities associated with exercise-induced pulmonary congestion in some patients [27]. Ex-CMR characterises the cardiovascular response to exercise; pulmonary function, CPET, metabolic measurements and symptoms complete its clinical interpretation.

### 3.3. Exercise Haemodynamics and Pressure–Flow Mismatch

Both exercise PCWP and its relationship to cardiac output help characterise HFpEF haemodynamics. The pressure–flow relationship helps distinguish a high-flow response from a disproportionate rise in pressure [28]. Concurrent measurements of pulmonary artery pressure, flow, resistance or compliance help identify pulmonary circulatory involvement [29]. Changes in stroke volume, cardiac output, atrial deformation and vascular area during exercise can help characterise functional responses associated with pressure–flow mismatch [29,30]. Ex-CMR complements invasive pressure measurements by relating these responses to chamber geometry and circulatory output [30].

### 3.4. Exercise as a Window onto Latent Physiological Abnormalities

Compensated resting haemodynamics may conceal mild impairments in diastolic, forward-flow or pulmonary vascular reserve. Symptom-provoking exercise increases venous return, heart rate, blood flow and vascular load, which can expose these latent mismatches. In Ex-CMR, the achieved workload, posture, acquisition timing, and image quality should match the clinical question. Imaging during exercise and after its cessation samples different physiological states. Recording the acquisition time distinguishes the exercise response from recovery.

## 4. Technical Foundations of Exercise CMR

### 4.1. Exercise Modalities and Equipment

An MR-compatible supine cycle ergometer allows real-time imaging during in-magnet exercise and minimises the delay between exercise and acquisition [31]. Exercise outside the magnet requires rapid transfer or repositioning for imaging. This approach reduces artefacts from ongoing movement but is more susceptible to haemodynamic recovery [32]. Handgrip exercise provides a stimulus with defined intensity, frequency and duration [33], although its responses cannot be directly extrapolated to dynamic whole-body exercise. Dobutamine increases heart rate and myocardial contractility [33] but does not fully reproduce the integrated physiological response to whole-body exercise. The stress modality therefore forms part of the interpretation of each reserve index [34].

Safety procedures should include screening for contraindications, electrocardiographic and symptom monitoring during exercise, blood pressure recording and predefined stopping criteria. The supervising team needs expertise in CMR, exercise testing and emergency care [35,36]. Exercise capacity, body habitus, musculoskeletal limitations and claustrophobia may affect whether patients complete the examination. Arrhythmias and motion artefacts can also reduce image quality and analysability for individual sequences [36,37]. Feasibility reports should describe participant flow and give the numbers and reasons for incomplete examinations or uninterpretable results [38]. Sequence-level counts of attempted and successful acquisitions, including failures among older patients and those with comorbidities, help assess applicability to routine HFpEF populations.

### 4.2. Real-Time Imaging and Acceleration Techniques

Higher heart rates, deeper breathing, rhythm irregularity and body motion during exercise limit conventional electrocardiographically gated breath-hold sequences [39]. Real-time free-breathing cine imaging underpins Ex-CMR assessment of chamber volumes, wall motion and atrial function [9,39]. Compressed sensing, parallel imaging and deep-learning reconstruction accelerate acquisition. Their effects on image quality and quantitative measurements need validation for each acquisition and reconstruction protocol [39,40]. Phase-contrast CMR measures aortic or pulmonary arterial flow, from which stroke volume and cardiac output can be derived by integrating flow curves [41]. Real-time phase-contrast imaging can measure exercise flow, provided the protocol accounts for respiratory and body motion, measurement-plane position, velocity encoding, background phase offsets, and arrhythmias [39,41]. Feature tracking derives atrial and ventricular deformation from cine images. Its results depend on image quality, analysis methods, and software platform [35,42]. LA-LAS instead uses geometric changes in atrial long-axis length throughout the cardiac cycle. Its measurement principle and numerical scale differ from feature-tracking strain, so diagnostic thresholds must be linked to the method used [9].

### 4.3. Measurable Parameters and Levels of Interpretation

Ex-CMR yields basic image measurements, derived functional indices, and model-based estimates. Basic measurements include chamber volumes, vascular cross-sectional area, velocity and signal intensity. Flow and deformation parameters are obtained through post-processing, including velocity integration and motion tracking [41,43]. Stroke volume, cardiac output, indexed atrial volumes and atrioventricular coupling indices are calculated from these measurements. CMR-estimated PCWP is a model output based on imaging variables [30]. Each level has distinct sources of error, beginning with the repeatability and accuracy of the basic measurements [41,43]. Derived indices should specify their formulae, units, inputs, segmentation rules and software versions. Model evaluation should report prediction error, calibration and validation methods, including subsequent assessment in independent populations. Diagnostic applications also require the basis for threshold selection and the performance at those thresholds [44]. An absolute exercise value is distinct from reserve. Reserve may be expressed as the exercise-minus-rest difference, percentage change from rest, or exercise-to-rest ratio; the definition should be explicit. Stroke volume derived from cine volume differences should also be distinguished from forward or net flow measured by phase-contrast imaging, particularly with regurgitation or shunting [41,43].

### 4.4. Minimum Requirements for Standardisation

Feasibility should be assessed at the level of the complete examination rather than the final analysable dataset alone. Reports should state the number of patients who started exercise, achieved the intended workload, completed each sequence, and contributed to each quantitative endpoint, together with reasons for termination or image exclusion. In the myocardial torsional-reserve study, 27 of 94 initially enrolled participants were excluded because of arrhythmia, slice mismatch, or triggering problems [17]. In a recent single-beat Ex-CMR reproducibility study, technically successful examinations were obtained in 136 of 141 participants; however, repeat imaging was available in only 15 healthy individuals, leaving reproducibility in patients with obesity, atrial fibrillation, pulmonary disease, or advanced HFpEF less certain [37]. Similarly, recovery-phase quantitative perfusion was analysable in 89 of 99 eligible participants after clinical exclusions, illustrating that feasibility varies by sequence and endpoint rather than by patient alone [45]. Protocol-level reports should therefore provide sequence-specific success rates, image-quality criteria, exclusion mechanisms, observer reproducibility, and, where possible, test–retest variability [31,32,35,39,40,41,42,43,46].

## 5. Exercise CMR in the Diagnosis of HFpEF

### 5.1. The Foundation Provided by Resting CMR and the Remaining Diagnostic Gap

The SCMR position paper by Leiner et al. describes the clinical value of CMR for assessing biventricular volumes, myocardial tissue, the pericardium and structural heart disease [8]. In patients with exertional dyspnoea, these measurements primarily support differential diagnosis. Left ventricular mass and atrial volumes characterise remodelling. Myocardial morphology, LGE distribution, native T1, T2 and ECV provide clues to amyloidosis, myocarditis, scar, hypertrophic cardiomyopathy and other infiltrative or storage diseases. Pericardial morphology, respiratory ventricular interdependence, and valve assessment help identify constrictive pericarditis and significant valvular disease [8,47]. Resting CMR establishes the structural and tissue findings against which exercise responses are interpreted. Normal resting findings cannot exclude exercise-induced filling-pressure abnormalities. Borlaug et al. found marked exercise PCWP elevation in some patients with exertional dyspnoea despite normal natriuretic peptide levels and resting haemodynamics [3]. The HFpEF-Stress study subsequently showed that real-time exercise CMR measurements of atrial function can reveal abnormalities that are less apparent at rest [9]. Exercise-specific evidence for differential diagnosis remains limited. Resting CMR remains the principal CMR examination for identifying infiltrative, inflammatory, hypertrophic, valvular, pericardial, or scar-related substrates that may mimic HFpEF. In particular, late gadolinium enhancement, native T1, extracellular volume, ventricular morphology, and pericardial features can support the recognition of cardiac amyloidosis, hypertrophic cardiomyopathy, constrictive pericarditis, or other structural causes of exertional symptoms [8,47]. The exercise literature provides supportive rather than definitive differentiation: Burrage et al. included patients with cardiac amyloidosis and showed distinct associations among myocardial energetics, ventricular reserve, and exercise-related pulmonary water signal [27], whereas Schulz et al. included hypertrophic cardiomyopathy as a comparator group in a recovery-phase quantitative perfusion study [45]. These studies demonstrate that Ex-CMR may add physiological context after resting CMR identifies a potential alternative substrate; they do not establish an exercise-CMR differential diagnostic algorithm for amyloidosis or hypertrophic cardiomyopathy.

### 5.2. Atrial and Volumetric Reserve Studies Referenced to Exercise Right Heart Catheterisation

In the 2021 HFpEF-Stress Trial, Backhaus et al. evaluated real-time free-breathing exercise CMR against PCWP measured by resting and exercise right heart catheterisation. Patients with exertional dyspnoea, preserved left ventricular ejection fraction, and echocardiographic signs of diastolic dysfunction were prospectively recruited. The final cohort comprised 68 participants: 34 with haemodynamically defined HFpEF and an equal number with non-cardiac dyspnoea. Real-time CMR measured left ventricular filling, left atrial emptying and long-axis deformation at rest and during exercise. These responses were compared with the invasive haemodynamic classification [9].

In this selected single-centre cohort, exercise LA-LAS was the best-performing CMR discriminator between HFpEF and non-cardiac dyspnoea. Its area under the receiver operating characteristic curve (AUC) increased from 0.82 at rest to 0.93 during exercise (*p* = 0.029) [9]. By contrast, neither total diastolic nor early diastolic left ventricular filling volume differed significantly between groups. Exercise atrial deformation may therefore be more sensitive to HFpEF-related dysfunction than some ventricular filling-volume indices. It may distinguish adaptations to stress in patients with similar resting chamber function.

Schulz et al. subsequently compared resting and exercise cardiac output measured by real-time phase-contrast CMR and invasive methods within the same programme. The study included 68 patients with exertional dyspnoea who underwent CMR and right heart catheterisation within 24 h. Measurements showed good agreement, with CMR tending to underestimate cardiac output [48]. Because the examinations were not simultaneous, differences in workload and physiological state may also have contributed to the variation between methods. The comparison supports real-time phase-contrast CMR for non-invasive quantification of exercise cardiac output.

The relationship between atrial and ventricular volumes adds diagnostic information. The left atrioventricular coupling index (LACI) developed by Backhaus et al. was higher in HFpEF at rest and during exercise than in non-cardiac dyspnoea. It correlated moderately with PCWP in the corresponding state (rest, r = 0.48; exercise, r = 0.55) [14]. Interpreting LACI alongside LA-LAS and cardiac output helps relate atrial deformation and atrioventricular filling to forward output. Its definition and phenotypic implications are discussed in Section 6.2.

### 5.3. Non-Invasive Estimation of Exercise Filling Pressure and Composite Diagnostic Indices

In a subsequent HFpEF-Stress analysis, Backhaus et al. applied existing PCWP equations to real-time CMR images acquired during free breathing at rest and during exercise. The equations used left ventricular mass and left atrial volume or area. Estimated PCWP correlated moderately with invasive measurements, with overestimation at rest and underestimation during exercise. The left atrial volume-based model identified HFpEF with an AUC of 0.81 during exercise, compared with 0.73 at rest. Exercise assessment also added diagnostic information in occult HFpEF, where filling pressure increased only during exertion [30]. Exercise images improved discrimination, particularly when resting haemodynamics were inconclusive.

Backhaus et al. subsequently developed a functional PCWP model incorporating left atrial reservoir strain and indexed left ventricular end-systolic volume. Derivation and validation cohorts included different pulmonary hypertension phenotypes. Resting CMR variables were used to estimate resting and exercise PCWP separately. The exercise-pressure model identified HFpEF with an AUC of 0.85, compared with 0.67 for a morphological model using left atrial volume and ventricular mass [49]. Including atrial function improved the model’s ability to link resting imaging findings with exercise haemodynamics.

Pressure models require separate assessment of numerical accuracy and the ability to identify elevated pressure. A high AUC can coexist with systematic pressure bias. Models that estimate continuous pressure values should report error and calibration; classification models require sensitivity and specificity at predefined thresholds. The two model types also use inputs acquired at different times. Applying an existing equation to exercise images and predicting exercise pressure from resting function address distinct diagnostic questions.

Ghanbari et al. proposed the myocardial dynamic index (MDI) using a work–volume loop framework. It combines mean resting and stress stroke volume index, the highest achieved exercise power, and left ventricular mass. The endpoint was cardiac dyspnoea, which is broader than invasively defined HFpEF. It included some patients with normal invasive pressures but abnormal resting imaging, as well as patients with mildly reduced ejection fraction. Among 93 patients with dyspnoea, MDI alone distinguished cardiac from non-cardiac dyspnoea with an AUC of 0.91. Adding MDI to a clinical and resting CMR model increased the AUC from 0.86 to 0.93 (*p* = 0.012) [50]. In this population, exercise performance and volumetric responses together added information beyond clinical and resting imaging assessment. As achieved power is an input, MDI incorporates both the cardiac response and the exercise load attained.

### 5.4. Complementarity with Established Diagnostic Pathways

Exercise echocardiography, cardiopulmonary exercise testing (CPET), invasive exercise haemodynamics, and Ex-CMR interrogate overlapping but non-identical aspects of exertional limitation. Exercise echocardiography can assess mitral inflow, tissue Doppler velocities, tricuspid regurgitation velocity, and cardiac output during exercise or early recovery; simultaneous invasive–echocardiographic studies have shown that its diagnostic interpretation is most informative when linked to exercise filling pressures [7]. CPET directly quantifies oxygen uptake, ventilatory efficiency, chronotropic response, and peripheral exercise limitation, whereas invasive exercise testing remains the reference method when the clinical question is whether PCWP, pulmonary artery pressure, or a pressure–flow relationship is abnormal during exertion [3,12,13,51]. H_2_FPEF and HFA-PEFF provide pre-test probability estimates, but both may remain indeterminate in patients whose abnormalities appear predominantly during exercise [4,5].

The HFpEF-Stress Trial provides an important physiological comparison platform because its participants underwent invasive exercise haemodynamic assessment, echocardiography, and real-time exercise CMR within the same research programme [9,10,11]. However, invasive testing and echocardiography were performed during the catheterisation protocol, whereas CMR was generally acquired on a separate day. The study therefore supports physiological associations between Ex-CMR measures and haemodynamic classification, but does not demonstrate that Ex-CMR provides incremental diagnostic value beyond stress echocardiography, H_2_FPEF, HFA-PEFF, or CPET in a same-day, independent, unselected referral cohort.

**Figure 1:**
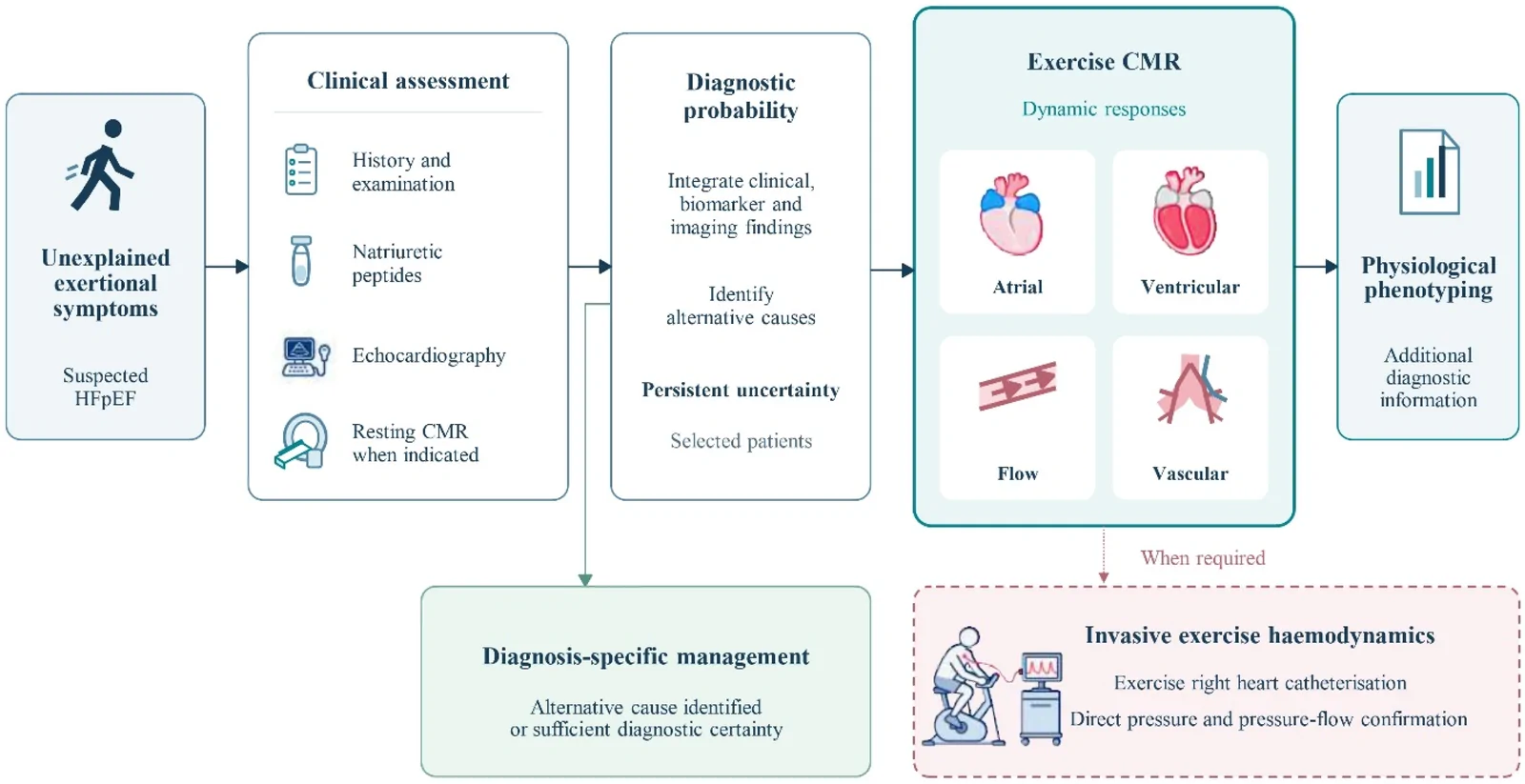
Proposed position of exercise CMR in evaluating exertional symptoms with suspected HFpEF. The proposed pathway is based on existing evidence and is intended for prospective evaluation. Clinical assessment, natriuretic peptides, echocardiography and, when indicated, resting CMR establish diagnostic probability and identify alternative causes. In selected patients with persistent uncertainty, Ex-CMR adds information about atrial, ventricular, flow and vascular responses. Invasive exercise haemodynamics provides direct pressure and pressure–flow confirmation when required.

The most defensible current role for Ex-CMR is therefore selective rather than replacement-based. In patients with persistent exertional symptoms, preserved LVEF, intermediate diagnostic probability after clinical assessment and standard testing, and inconclusive or technically limited echocardiography, Ex-CMR can characterise dynamic chamber volumes, atrial deformation, forward flow, and vascular responses within one examination [4,5,9,10,11,31,52]. When a definitive pressure-based diagnosis, pulmonary pressure measurement, or pressure–flow assessment is required, invasive exercise haemodynamics remains necessary [3,51]. Figure 1 illustrates the proposed position of Ex-CMR within this selective diagnostic pathway.

### 5.5. Interpretation of Diagnostic Performance and Limits of the Evidence

The current diagnostic evidence has a clear hierarchy. The most direct evidence comes from the HFpEF-Stress programme, in which real-time Ex-CMR measures were interpreted against invasive resting and exercise PCWP [9,10,11,14,30,48]. Exercise LA-LAS, LACI, CMR-derived cardiac output, and CMR-estimated PCWP should therefore be interpreted as related signals obtained from overlapping or partially overlapping cohorts, rather than as independently replicated diagnostic tests. The functional PCWP model similarly extends this programme of work by incorporating left atrial functional variables, but remains a prediction model requiring external calibration in clinically representative HFpEF populations [49]. Schuster et al. provided complementary invasive reference evidence for latent pulmonary vascular disease, although the pulmonary vascular phenotype and its threshold were study-specific [19]. By contrast, the myocardial dynamic index and biventricular reserve studies provide independent but exploratory evidence from broader cardiac-dyspnoea or HFpEF-spectrum populations; neither used invasive haemodynamic HFpEF classification as the sole target condition [50,53].

**Table 1:** Design and Evidence Relationships of Exercise CMR Studies.

Study and Source	Population and Reference	CMR Acquisition Stage	Principal Contribution	Evidence Relationships and Interpretation
HFpEF-Stress primary study [9]	68 participants: 34 HFpEF and 34 NCD; resting and exercise RHC	Real-time imaging at rest and during supine exercise	LA-LAS AUC: 0.82 at rest and 0.93 during exercise	Selected single-centre cohort used in several subsequent analyses
LACI [14] and cardiac output [48]	68 participants in each analysis; RHC comparison	Volumes or real-time phase-contrast flow during exercise	Association of the atrioventricular volume ratio with PCWP; measurement of forward output	HFpEF-Stress secondary analyses; CMR and RHC were not simultaneous
Morphological PCWP model [30]; functional model [49]	Invasive pressure reference; the latter included derivation and validation cohorts	Morphological model: resting and exercise images; functional model: resting inputs only	Exercise-image model AUC 0.81; resting-function prediction model AUC 0.85	Input timing and comparator models differ; discrimination assessed within each study
myocardial dynamic index [50]	93 patients with dyspnoea: 66 cardiac and 27 NCD	Resting and post-exercise volumes, combined with maximal exercise power	MDI alone: AUC 0.91; base model 0.86 → 0.93 with MDI	Broad cardiac-dyspnoea endpoint; possible cohort overlap with contemporaneous volume studies
Ghanbari biventricular reserve [53]	140 participants: 40 healthy, 27 NCD and 73 within a study-defined HFpEF spectrum	10–20 s after supine exercise cessation	Four volume-reserve measures combined into six response patterns	Multicentre recruitment, single CMR centre; stage B had a study-specific definition
Morales torsional reserve [17]	94 initially enrolled; 67 analysable	Single-beat tagging at rest and after exercise	Reduced torsional augmentation in HFpEF	27 excluded for acquisition or rhythm problems; untwisting not directly measured
HFpEF-Stress right-heart and pulmonary artery analyses [19,20,21]	67, 68 and 63 participants, respectively; invasive haemodynamic classification	Real-time cine and pulmonary artery flow during exercise	Right ventricular output and adaptation to load; changes in pulmonary arterial area	Shared source cohort; area-based indices are compliance surrogates
Petit simultaneous CMR/RHC [23]	16 HFpEF, 7 with impaired relaxation and 8 healthy controls	Exercise CMR with simultaneous central pressure measurements	Right ventricular reserve and pressure–flow responses; acute pharmacological assessment	Small mechanistic study assessing the acute response to a single dose
Burrage multimodal study [27]	43 participants, including 14 with HFpEF	Resting PCr/ATP; chamber function during 20 W exercise; pre-/post-exercise lung water	Associations of energetic status with biventricular responses and lung-water changes	Cross-group associations between resting energetics and exercise responses
Schulz quantitative perfusion [45]	108 recruited; 99 assessed for feasibility and 89 analysable	Approximately 45–60 s after exercise cessation	Recovery-phase quantitative perfusion in HFpEF, HCM and NCD	Recovery-phase perfusion; at least one paired slice required for analysis
HFpEF-Stress follow-up [11,14,21]	68 or 63 participants by index; 24-month or four-year follow-up	Resting and exercise indices related to subsequent events	Atrioventricular coupling, atrial and pulmonary arterial indices associated with events	Endpoints predominantly cardiovascular hospitalisation; cohorts and event sets overlap

Abbreviations: NCD, non-cardiac dyspnoea; RHC, right heart catheterisation; HCM, hypertrophic cardiomyopathy. AUC values refer to each study’s population, endpoint and acquisition timing.

Multiple HFpEF-Stress analyses draw on a shared source cohort, providing complementary physiological observations rather than independent replication. A high AUC describes discrimination within that cohort; clinical application also requires sensitivity, specificity and calibration at predefined thresholds. Independent validation should include consecutive referrals across acquisition platforms, exercise protocols and rhythms. Posture, workload and acquisition timing should match the reference examination. Diagnostic-pathway evaluation should include unanalysable examinations and assess incremental value beyond H_2_FPEF, HFA-PEFF, stress echocardiography and CPET.

A priority group comprises patients with intermediate diagnostic probability after routine evaluation who need further assessment of cardiac reserve. Prospective pathway studies should test whether Ex-CMR improves diagnostic certainty and referral decisions for exercise right heart catheterisation.

## 6. Physiological Phenotyping of HFpEF with Exercise CMR

### 6.1. A Physiological Phenotyping Framework Based on Exercise Reserve

Patients with HFpEF may have similar symptoms and resting ejection fractions but different physiological constraints on exercise capacity. Exercise CMR characterises this heterogeneity by assessing atrial function and atrioventricular coupling, biventricular volume and output reserve, right ventricular–pulmonary circulatory adaptation, and systemic arterial responses. Resting energetics and post-exercise perfusion add information on metabolism and microvascular function. These abnormalities can coexist in an individual and form the basis of research into physiological phenotypes. Uniform clinical thresholds and treatment-selection rules remain to be established. Table 2 summarises measurement definitions and interpretive considerations.

### 6.2. Left Atrial Reserve, Atrioventricular Coupling and Modifying Factors

Left atrial reserve is the capacity to accommodate increased pulmonary venous return and support ventricular filling through coordinated reservoir, conduit and active contractile responses to exercise. Impaired reserve may involve insufficient changes in deformation, emptying or volume during exercise, together with atrial enlargement. Ex-CMR describes these responses using LA-LAS, atrial ejection fraction, volume changes and LACI. Deformation and emptying primarily describe atrial function, whereas LACI characterises the atrioventricular volume relationship at a specified cardiac phase. Assessing these measures together may reveal impaired adaptation that resting morphology does not fully capture.

In the HFpEF-Stress Trial, exercise accentuated differences in atrial function that were less apparent at rest [9]. Physiologically, reduced atrial deformation reflects limited adaptation to increased venous return and filling load. Atrial dysfunction, ventricular diastolic properties, and loading conditions can each contribute. Assessing LA-LAS with atrial emptying, ventricular volumes, and forward output relates atrial deformation to the wider atrioventricular response.

LACI is calculated as left atrial volume divided by left ventricular volume at the same ventricular end-diastolic phase, multiplied by 100%. In the HFpEF-Stress cohort, resting and exercise LACI were 45.7% and 45.7% in HFpEF, versus 31.6% and 27.9% in non-cardiac dyspnoea. Between-group differences were significant in both states (*p* < 0.001) [14]. An elevated LACI can reflect greater residual atrial volume, smaller ventricular filling volume, or both. Examining the component volumes alongside the ratio helps explain the limited atrioventricular adjustment suggested by these exercise responses.

Resting atrial geometry also helps interpret the exercise response. In a cross-sectional three-dimensional shape analysis, Backhaus et al. identified local atrial roof enlargement in patients whose filling pressure rose only during exercise. Elevated resting pressure was associated with more global enlargement [16]. These spatial differences suggest that regional atrial geometry may add information beyond total atrial volume.

Comorbidities and fat distribution may modify atrial responses to exercise. In 68 HFpEF-Stress participants, Schulz et al. found more overt HFpEF with higher epicardial adipose tissue (EAT) burden (64% versus 37%). Higher EAT was associated with impaired atrial strain at rest and during exercise, and with impaired ventricular strain during exercise. Biventricular volumes and ventricular mass did not differ significantly [26]. By contrast, a study of 32 patients with obesity and HFpEF found no significant association between resting MRI-derived ventricular EAT and seated exercise filling pressure or exercise capacity [54]. These studies assessed different anatomical regions, exercise postures, and patient populations. These differences matter when interpreting associations between EAT and exercise phenotypes.

Atrial and ventricular responses to exercise also differ by sex. A stratified HFpEF-Stress analysis found lower exercise right ventricular stroke volume index in women than men with HFpEF (38.1 versus 50.4 mL/m^2^; *p* = 0.011). Rest-to-exercise changes in atrial ejection fraction were −2.7 and +2.5 percentage points, respectively (*p* = 0.020). Exercise left ventricular filling volume was also lower in women [15]. These small-sample associations support considering body size, sex, and loading conditions when interpreting atrial and ventricular reserve.

### 6.3. Left Ventricular and Biventricular Volumetric and Mechanical Reserve

Ventricular reserve concerns the capacity to increase stroke volume through changes in end-diastolic and end-systolic volumes and myocardial deformation, coordinated between the ventricles. Abnormalities include insufficient filling-volume recruitment, limited end-systolic volume reduction, or discordant ventricular responses to stress. Preserved resting ejection fraction does not necessarily indicate adequate output reserve during exercise. Comparing biventricular volumes and mechanics at rest, during exercise or immediately afterwards helps distinguish patterns of impaired reserve.

Ghanbari et al. retrospectively analysed a prospectively recruited multicentre cohort. It comprised 40 healthy controls, 27 patients with non-cardiac dyspnoea and 73 participants within a study-defined HFpEF spectrum. All Ex-CMR examinations took place at one imaging centre, where cine images were acquired 10–20 s after supine cycling stopped. Left ventricular end-diastolic volume reserve was limited across HFpEF subgroups, whereas insufficient end-systolic volume reduction occurred mainly in stage C. Right ventricular reserve was more impaired with coexisting pulmonary hypertension. Combinations of four biventricular volume-reserve parameters defined six response patterns [53]. This cross-sectional analysis distinguishes patterns of ventricular filling and emptying within the study population.

Volume reserve was defined as (post-exercise volume − resting volume)/resting volume × 100%. The ventricular efficiency index subtracted percentage end-systolic volume change from percentage end-diastolic volume change; the biventricular index summed the two ventricular indices [53]. Here, efficiency denotes volumetric adaptation, not myocardial energy efficiency. The study-defined stage B included symptomatic patients with exertional dyspnoea, normal resting and exercise filling pressures, and CMR abnormalities. This differs from guideline stage B, or pre-heart failure, which excludes current or previous heart failure symptoms [53,55].

Morales et al. used myocardial tagging to assess systolic torsional augmentation, a mechanical measure of reserve. Myocardial torsional reserve was defined as post-exercise torsion divided by resting torsion; diastolic untwisting was not directly assessed. Of 94 initially enrolled participants, 27 were excluded for arrhythmias, slice mismatch, or triggering problems. Among 67 analysable participants, values were 1.1 ± 0.1 and 1.2 ± 0.2 in exercise-induced and overt HFpEF, respectively. These were lower than in healthy controls (1.7 ± 0.4) and non-cardiac dyspnoea (1.6 ± 0.4) [17]. The lower torsional augmentation indicates impaired mechanical reserve despite preserved resting ejection fraction.

Other mechanical measures assess how filling and ejection are coordinated. Using resting cine images, Backhaus et al. identified lower longitudinal haemodynamic forces, systolic peaks and impulses in HFpEF, together with abnormalities related to late diastolic filling [18]. Edlund et al. studied 13 healthy volunteers and 22 heart failure patients across ejection-fraction categories. Exercise changed the ventricular–atrial short-axis area difference in opposite directions in the two groups. It decreased in heart failure but increased in controls, suggesting less favourable geometry for diastolic filling [56]. Resting haemodynamic forces and exercise changes in the geometric component of hydraulic force describe different aspects of filling and ejection.

### 6.4. Right Ventricular–Pulmonary Circulatory Reserve

Right ventricular–pulmonary circulatory reserve describes the capacity of the right ventricle to accommodate increased pulmonary flow and changing vascular load during exercise. Abnormalities may include insufficient stroke volume augmentation, limited longitudinal contractile reserve, or a mismatch between pulmonary arterial expansion and flow demand, even when ejection fraction is preserved. Ex-CMR measures ventricular volumes, deformation, forward flow, and pulmonary arterial area changes together. These measurements can reveal right-heart limitations that are less apparent at rest. Adding invasive pressure measurements relates these imaging findings to pulmonary vascular load.

Among 67 HFpEF-Stress participants, Schuster et al. used a study-specific definition of latent pulmonary vascular disease: exercise pulmonary vascular resistance ≥1.74 Wood units. They compared 24 participants meeting this definition with the remaining 43. Those meeting the definition had lower exercise right ventricular longitudinal function, stroke volume and peak flow, together with reduced atrial filling and left ventricular cardiac index. The right ventricular peak-flow index discriminated this haemodynamic grouping better during exercise than at rest (AUC 0.89 versus 0.73; *p* = 0.004) [19]. The concurrent reductions in right ventricular output, atrial filling and left ventricular cardiac index relate pulmonary circulatory limitation to the overall cardiac response to exercise.

Wolter et al. examined how the right ventricle adapted to exercise load in 34 patients with HFpEF and 34 with non-cardiac dyspnoea. Pulmonary artery pressure and CMR volume measurements were combined to derive surrogates of effective arterial elastance and end-systolic elastance. Resting afterload and contractility indices were higher in HFpEF, but relative contractility augmentation during exercise was smaller. Reserve was most limited in patients with elevated resting filling pressure [20]. The exercise increases in the afterload-to-contractility ratio, Ea/Ees, did not reach statistical significance in HFpEF (*p* = 0.078). The main finding was limited exercise augmentation of contractility despite higher resting contractility and afterload.

Schulz et al. assessed pulmonary arterial responses to exercise. In 63 participants with normal resting right ventricular ejection fraction, real-time phase-contrast imaging measured main pulmonary artery area pulsatility and pulsatility normalised to right ventricular stroke volume. These indices are imaging surrogates of pulmonary arterial compliance that do not include pressure measurements. Resting values were similar in HFpEF and non-HFpEF groups, but exercise area pulsatility was lower in HFpEF (13% versus 20%; *p* < 0.001). The normalised index was also lower, and reduced exercise indices were associated with subsequent cardiovascular events [21]. Exercise revealed abnormal arterial expansion despite preserved resting right ventricular ejection fraction.

Serial imaging can track right ventricular changes over time. Backhaus et al. repeated CMR in HFpEF-Stress participants. Resting and exercise right ventricular long-axis strain declined in the HFpEF subgroup without interatrial shunt implantation. No corresponding change occurred in non-cardiac dyspnoea [22]. Because strain depends on both ventricular function and loading conditions, its serial measurement tracks their combined effects at rest and during exercise.

Petit et al. combined exercise CMR with simultaneous invasive pressure measurements to relate right ventricular volume responses to pulmonary circulatory load. HFpEF and concentric remodelling with impaired relaxation were associated with less favourable exercise pulmonary pressure–flow responses and lower reserve in the right ventricular end-systolic pressure–volume ratio. The latter was associated with peak oxygen uptake [23]. This mechanistic study suggests that limited right ventricular compensation can emerge even when resting abnormalities are mild. The intervention findings are discussed in Section 7.

### 6.5. Systemic Arterial Load

Aortic elasticity and the blood pressure response to exercise influence left ventricular ejection and diastolic filling. Greater aortic stiffness can impair buffering of pulsatile flow, increase ejection load and alter ventricular–arterial coordination. CMR assesses systemic arterial load using aortic pulse wave velocity (PWV), vascular area changes, and distensibility calculated with pulse pressure. Studies have examined resting stiffness in relation to abnormal exercise haemodynamics, as well as changes in aortic distensibility after acute exercise. The measurements address different aspects of arterial function.

Schulz et al. analysed resting aortic PWV in 48 HFpEF-Stress participants. Values were 7.0, 10.0 and 11.0 m/s in non-cardiac dyspnoea, occult HFpEF and overt HFpEF, respectively. Both HFpEF groups had higher values than non-cardiac dyspnoea (*p* = 0.005 and *p* = 0.01, respectively) [24]. These resting measurements characterise arterial stiffness in patients with exercise filling-pressure abnormalities.

Orizu et al. assessed recovery-phase aortic distensibility in twenty-four heart failure patients and 26 controls. Aortic imaging was performed before and approximately 1 min after submaximal supine cycling, with distensibility calculated using non-invasive pulse pressure. Distensibility decreased in the overall heart failure group, mainly driven by the study-defined reduced-ejection-fraction subgroup. No significant change occurred in the HFpEF subgroup of 10 patients [25].

Evidence on systemic arterial load currently comes mainly from associations with resting stiffness. To determine whether these measurements add independent phenotypic information, aortic area and velocity changes need to be related to blood pressure, ventricular output and filling at the same stage.

### 6.6. Energetics, Perfusion and Multiparametric Integration

Myocardial energetics, perfusion and lung-water imaging examine related components of exercise intolerance. The phosphocreatine-to-ATP ratio (PCr/ATP) reflects myocardial high-energy phosphate status. Quantitative myocardial blood flow and perfusion reserve describe perfusion under different loading conditions, while lung proton-density imaging assesses exercise-related lung-water signal changes. Combining these measurements with atrial and biventricular function helps relate energy status and blood supply to mechanical responses and pulmonary congestion.

Burrage et al. studied 11 healthy controls, 9 patients with type 2 diabetes without HFpEF, 14 with HFpEF, and 9 with cardiac amyloidosis. The protocol combined resting phosphorus spectroscopy with measurements of chamber function during 20 W exercise and lung proton-density imaging before and after exercise. Lung proton-density signal includes both intravascular and extravascular water. PCr/ATP decreased across these four groups; median values were 2.15 in healthy controls and 1.66 in HFpEF. Across groups, lower resting PCr/ATP was associated with poorer left ventricular diastolic reserve, smaller right ventricular ejection-fraction augmentation, and greater exercise-induced lung-water signal increases. Lung proton-density signal increased by 4.4% in HFpEF, with no significant change in healthy controls [27]. Lower resting energetic status was thus associated with exercise-related ventricular dysfunction and transient congestion.

Quantitative perfusion imaging measures myocardial blood flow after exercise. Schulz et al. prospectively recruited 108 participants. After exclusion of 9 participants with obstructive coronary disease or reduced ejection fraction, 99 underwent feasibility assessment. Perfusion images were acquired approximately 45–60 s after exercise cessation. Feasibility required at least one paired resting and post-exercise slice suitable for quantitative analysis. This criterion was met by 89 participants: 34 with hypertrophic cardiomyopathy, 34 with HFpEF and 21 with non-cardiac dyspnoea. Blood flow and perfusion reserve were lower in hypertrophic cardiomyopathy than in the other groups, and peak exercise heart rate independently predicted both measures [45]. The study supports quantitative perfusion imaging during recovery and relates the measured response to the physiological load achieved.

Composite models combine individual imaging measures with exercise performance. In a work–volume loop study of 120 participants, Ghanbari et al. combined resting and post-exercise left ventricular end-diastolic and end-systolic volumes with maximal exercise power. These inputs generated trapezoidal loops and associated geometric indices. With exercise power rather than ventricular pressure on the vertical axis, loop area is a model-derived geometric measure, not myocardial stroke work. Model-defined compliance reserve was impaired in exercise-induced and stage C HFpEF, whereas power-adjusted contractile reserve was higher in exercise-induced HFpEF and lower in stage C [57]. Accounting for workload can distinguish responses that appear similar when only absolute volume changes are considered.

Multiparametric assessment examines how reserve abnormalities coexist and what each measurement adds. The six biventricular volume-reserve patterns were constructed from combinations of reference thresholds [53]. Clinical evaluation of such patterns requires independent assessment of reproducibility and incremental value. One question is whether energetics, perfusion, and lung-water signals explain differences in symptoms among patients with similar volumetric responses. Another is whether they improve prediction of outcomes or treatment response.

## 7. Prognostic Value and Treatment Monitoring

Current prognostic evidence is preliminary and derives mainly from related analyses within the HFpEF-Stress programme. In 68 participants, higher resting and exercise LACI were associated with non-elective cardiovascular hospitalisation over 24 months [14]. In the final HFpEF-Stress follow-up report, 23 cardiovascular hospitalisations occurred over four years, and impaired atrial functional measures were associated with subsequent events [11]. Schulz et al. subsequently reported associations between impaired exercise pulmonary artery pulsatility or stroke-volume-normalised capacitance and cardiovascular events in 63 participants; however, the hazard ratios were imprecise, including a hazard ratio of 9.6 with a 95% confidence interval of 1.2–75.8 [21]. These findings suggest that impaired atrial and pulmonary vascular reserve may identify clinically relevant risk, but they should be regarded as hypothesis-generating until externally validated against established predictors, including clinical risk factors, natriuretic peptides, CPET variables, and invasive haemodynamics. Importantly, the LACI, atrial functional, and pulmonary artery compliance analyses originate largely from the same specialist research programme and should therefore be interpreted as complementary observations rather than independent prognostic replications [11,14,21].

The evidence for treatment monitoring is more limited. No prospective study has used Ex-CMR as a primary endpoint to assess SGLT2 inhibitors, GLP-1 receptor agonists, weight-loss interventions, mineralocorticoid receptor antagonists, or structured exercise training in HFpEF. Petit et al. combined exercise CMR, simultaneous central haemodynamics, and cyclic guanosine monophosphate measurements before and after a single 50 mg sildenafil dose in a small mechanistic cohort; acute sildenafil did not improve circulating cyclic guanosine monophosphate concentration or right ventricular performance [23]. This study demonstrates how Ex-CMR can be integrated into mechanistic experiments, but it does not establish a treatment-response biomarker or support therapeutic selection.

Accordingly, Ex-CMR is currently best positioned as a research tool for physiological enrichment and mechanistic endpoint development. Candidate measures include exercise LA-LAS, LACI, biventricular volume reserve, exercise cardiac output, pulmonary artery pulsatility, myocardial energetics, and lung-water signals [11,14,21,27,48,53]. Future intervention studies should pre-specify a limited set of reproducible Ex-CMR endpoints, standardise workload and acquisition timing, and test whether changes in imaging measures predict patient-centred outcomes rather than only changes in physiological severity.

## 8. Current Challenges and Future Directions

The principal challenge for Ex-CMR is not the absence of measurable physiological signals, but the absence of a standardised, clinically validated pathway for obtaining and interpreting them. Heart-rate acceleration, respiratory motion, arrhythmia, body movement, and vessel displacement constrain real-time cine and phase-contrast measurements during exercise. Supine cycling also changes venous return and loading conditions relative to upright exercise, while recovery-phase imaging captures a changing physiological state rather than true peak exercise. Real-time acquisition, accelerated reconstruction, and automated analysis can shorten the effective acquisition window, but each method requires validation for its effect on volumetric, flow, and deformation measurements [31,32,35,37,39,40,41,42,43,52].

Implementation also requires MR-compatible exercise equipment, trained CMR technologists, physician supervision, exercise-physiology expertise, monitoring capacity, protocol-specific safety procedures, and time for real-time image planning and post-processing. Individualised exercise protocols may improve workload achievement, but they add operational complexity [46,52]. No study has yet established the cost-effectiveness, optimal scan duration, throughput, or learning curve of Ex-CMR in routine HFpEF referral pathways. These factors are particularly relevant because the patients most likely to need further physiological characterisation are often older and may have obesity, frailty, atrial fibrillation, musculoskeletal limitations, chronic kidney disease, or pulmonary disease.

Reproducibility and reference values remain important evidence gaps. Feature tracking and phase-contrast measurements depend on image quality, contouring strategy, software implementation, background phase correction, and exercise protocol [41,42,43]. Ghanbari et al. showed high reproducibility of single-beat free-breathing exercise CMR measurements, but the test–retest sample was limited to 15 healthy participants [37]. Schweitzer et al. subsequently reported sex- and age-stratified exercise CMR reference ranges in 161 healthy participants, providing a useful starting point for interpretation; however, these ranges require validation across scanners, ethnic groups, exercise protocols, and clinical populations before they can define HFpEF thresholds [58].

Most evidence comes from selected cohorts, and HFpEF-specific clinical thresholds remain limited. External validation needs to address both issues. HFpEF-Stress imposed restrictions on rhythm during examination and on selected pulmonary, coronary and valvular diseases [9,11]. Multicentre studies enrolling consecutive referrals across rhythms, body sizes and comorbidity burdens would test applicability to routine practice. Shared acquisition and analysis rules would support independent validation against the diagnostic endpoints in Section 5.5. They would also allow evaluation of physiological patterns such as the volumetric combinations described in Section 6.3. Cross-sectional patterns describe between-patient differences; longitudinal data are needed to assess individual trajectories. Evaluation should include external calibration and unanalysable examinations, as well as the costs and accessibility of the equipment, personnel and analysis required.

The patients most likely to benefit from Ex-CMR are those with persistent exertional symptoms, preserved LVEF, intermediate pre-test probability after H_2_FPEF or HFA-PEFF assessment, and unresolved diagnostic uncertainty after routine echocardiography, natriuretic peptide testing, and, where appropriate, CPET [4,5,7]. In this group, Ex-CMR may provide integrated information about atrial, biventricular, flow, and vascular reserve and help determine whether invasive exercise haemodynamics is warranted. It should not be used to replace invasive assessment when direct confirmation of PCWP, pulmonary artery pressure, or pressure–flow relationships is required [3,51].

Future work should prioritise measures that answer a defined clinical question and can be reproduced under realistic exercise conditions. Real-time cine imaging, phase-contrast flow, and chamber deformation analysis form the most practical core dataset. Additional modules—such as 4D flow, quantitative perfusion, lung-water imaging, phosphorus spectroscopy, and oxygen-sensitive CMR—should be added only when they provide information not available from the core examination. In HFpEF, phosphorus spectroscopy may help link impaired myocardial energetic reserve with exercise-related pulmonary congestion and biventricular reserve [27]. Blood oxygen level-dependent T2 mapping has also been associated with invasively measured central venous oxygen saturation in cardiovascular patients, indicating a potential route towards non-invasive assessment of oxygen-related physiology; however, it has not yet been validated as an exercise-HFpEF diagnostic tool [59]. The immediate priority is multicentre validation of a small number of core Ex-CMR measures against invasive exercise haemodynamics and patient-centred outcomes.

## 9. Conclusions

Ex-CMR adds measurements of atrial, biventricular and forward-output reserve to resting morphological assessment in HFpEF. Studies using invasive haemodynamic references support the diagnostic potential of exercise LA-LAS. Atrioventricular coupling and right ventricular–pulmonary circulatory responses characterise overlapping reserve abnormalities. Resting tissue characterisation and energetics, together with recovery-phase perfusion and lung-water signals, help interpret the mechanisms underlying these abnormalities. Current evidence supports an adjunctive role in the physiological assessment of selected patients at specialist centres. Standardised protocols and independent multicentre evaluation are needed to test whether these measurements improve diagnostic decisions and patient outcomes.

## Figures and Tables

**Table 2:** Representative Measures and Interpretive Considerations for Physiological Phenotypes.

Physiological Dimension	Representative Measures and Definitions	Physiological Interpretation	Key Qualifications And Evidence Stage
Left atrium and atrioventricular coupling	LA-LAS: long-axis length change; LACI = atrial volume/ventricular volume at the same ventricular end-diastolic phase × 100%	Atrial reservoir response and coordination of atrioventricular volumes	LA-LAS differs from feature-tracking strain; influenced by rhythm, load and ventricular filling; invasive-reference evidence available [9,14]
Biventricular volumes and mechanics	ΔV% = (post-exercise V − resting V)/resting V × 100%; torsional reserve = post-exercise/resting torsion	Filling recruitment, end-systolic volume adjustment and torsional augmentation	Specify direction of change and timing; volumetric efficiency is not energetic efficiency; research phenotyping [17,53]
Right ventricle and pulmonary circulation	Ventricular volumes, stroke volume and peak flow; pulmonary artery area pulsatility = (maximum area − minimum area)/minimum area × 100%	Adaptation of right ventricular output to pulmonary circulatory load	Pressure-free compliance surrogates; PVR and pressure–flow relationships require haemodynamic measurements [19,20,21]
Systemic arterial load	Aortic PWV; distensibility calculated from area change and pulse pressure	Arterial stiffness and ventricular–arterial interaction	Distinguish resting PWV from post-exercise distensibility; limited dynamic evidence in HFpEF [24,25]
Myocardial energetics and lung water	Resting PCr/ATP; pre-/post-exercise lung proton-density signal change	Links between energetic status, functional reserve and pulmonary congestion	Resting energetic status; lung signal includes intravascular and extravascular water; mechanistic evidence [27]
Myocardial perfusion	Recovery-phase quantitative myocardial blood flow; perfusion reserve = stress/resting blood flow	Blood supply response to stress	Influenced by peak heart rate, acquisition delay and analysable slices; no HFpEF-specific phenotyping thresholds [45]

Abbreviations: V, ventricular volume; PVR, pulmonary vascular resistance.

## Data Availability

No new data were created or analyzed in this study. Data sharing is not applicable to this article.

## Supplementary Materials

The following supporting information can be downloaded at: https://www.mdpi.com/article/10.3390/diagnostics16213462/s1, Table S1A: Search strategy and eligibility criteria; Table S1B: Database-specific search strings; Table S2: Structured appraisal of the primary clinical and technical evidence for exercise cardiovascular magnetic resonance in heart failure with preserved ejection fraction.

## References

[1] Owan T.E., Hodge D.O., Herges R.M., Jacobsen S.J., Roger V.L., Redfield M.M. (2006). Trends in Prevalence and Outcome of Heart Failure with Preserved Ejection Fraction. N. Engl. J. Med..

[2] Dunlay S.M., Roger V.L., Redfield M.M. (2017). Epidemiology of heart failure with preserved ejection fraction. Nat. Rev. Cardiol..

[3] Borlaug B.A., Nishimura R.A., Sorajja P., Lam C.S.P., Redfield M.M. (2010). Exercise Hemodynamics Enhance Diagnosis of Early Heart Failure With Preserved Ejection Fraction. Circ. Heart Fail..

[4] Reddy Y.N.V., Carter R.E., Obokata M., Redfield M.M., Borlaug B.A. (2018). A simple, evidence-based approach to help guide diagnosis of heart failure with preserved ejection fraction. Circulation.

[5] Pieske B., Tschöpe C., De Boer R.A., Fraser A.G., Anker S.D., Donal E., Edelmann F., Fu M., Guazzi M., Lam C.S.P. (2019). How to diagnose heart failure with preserved ejection fraction: The HFA–PEFF diagnostic algorithm: A consensus recommendation from the Heart Failure Association (HFA) of the European Society of Cardiology (ESC). Eur. Heart J..

[6] McDonagh T.A., Metra M., Adamo M. (2021). 2021 ESC Guidelines for the diagnosis and treatment of acute and chronic heart failure. Eur. Heart J..

[7] Obokata M., Kane G.C., Reddy Y.N.V., Olson T.P., Melenovsky V., Borlaug B.A. (2017). Role of Diastolic Stress Testing in the Evaluation for Heart Failure With Preserved Ejection Fraction: A Simultaneous Invasive-Echocardiographic Study. Circulation.

[8] Leiner T., Bogaert J., Friedrich M.G., Mohiaddin R., Muthurangu V., Myerson S., Powell A.J., Raman S.V., Pennell D.J. (2020). SCMR Position Paper (2020) on clinical indications for cardiovascular magnetic resonance. J. Cardiovasc. Magn. Reson..

[9] Backhaus S.J., Lange T., George E.F., Hellenkamp K., Gertz R.J., Billing M., Wachter R., Steinmetz M., Kutty S., Raaz U. (2021). Exercise Stress Real-Time Cardiac Magnetic Resonance Imaging for Noninvasive Characterization of Heart Failure With Preserved Ejection Fraction: The HFpEF-Stress Trial. Circulation.

[10] Backhaus S.J., Rösel S.F., Schulz A., Lange T., Hellenkamp K., Gertz R.J., Wachter R., Steinmetz M., Kutty S., Raaz U. (2022). RT-CMR Imaging for Noninvasive Characterization of HFpEF. JACC Cardiovasc. Imaging.

[11] Backhaus S.J., Schulz A., Lange T., Schmidt-Schweda L.S., Evertz R., Kowallick J., Hasenfuß G., Schuster A. (2024). Real-time cardiovascular magnetic resonance imaging for non-invasive characterisation of heart failure with preserved ejection fraction: Final outcomes of the HFpEF stress trial. Clin. Res. Cardiol..

[12] Borlaug B.A., Olson T.P., Lam C.S.P., Flood K.S., Lerman A., Johnson B.D., Redfield M.M. (2010). Global Cardiovascular Reserve Dysfunction in Heart Failure With Preserved Ejection Fraction. J. Am. Coll. Cardiol..

[13] Borlaug B.A., Jaber W.A., Ommen S.R., Lam C.S.P., Redfield M.M., Nishimura R.A. (2011). Diastolic relaxation and compliance reserve during dynamic exercise in heart failure with preserved ejection fraction. Heart.

[14] Backhaus S.J., Lange T., Schulz A., Evertz R., Frey S.M., Hasenfuß G., Schuster A. (2023). Cardiovascular magnetic resonance rest and exercise-stress left atrioventricular coupling index to detect diastolic dysfunction. Am. J. Physiol. Heart Circ. Physiol..

[15] Schulz A., Lange T., Evertz R., Kowallick J.T., Hasenfuß G., Backhaus S.J., Schuster A. (2023). Sex-Specific Impairment of Cardiac Functional Reserve in HFpEF. JACC Adv..

[16] Backhaus S.J., Nasopoulou A., Lange T., Schulz A., Evertz R., Kowallick J.T., Hasenfuß G., Lamata P., Schuster A. (2024). Left Atrial Roof Enlargement Is a Distinct Feature of Heart Failure With Preserved Ejection Fraction. Circ. Cardiovasc. Imaging.

[17] Morales M.A., Ghanbari F., Rodriguez J., Gopal D.M., Waxman A.B., Manning W.J., Nezafat R. (2026). Quantification of exercised-induced myocardial deformation with exercise CMR. J. Cardiovasc. Magn. Reson..

[18] Backhaus S.J., Uzun H., Rösel S.F., Schulz A., Lange T., Crawley R.J., Evertz R., Hasenfuß G., Schuster A. (2022). Hemodynamic force assessment by cardiovascular magnetic resonance in HFpEF: A case-control substudy from the HFpEF stress trial. eBioMedicine.

[19] Schuster A., Schulz A., Lange T., Evertz R., Hartmann F., Kowallick J.T., Hellenkamp K., Uecker M., Seidler T., Hasenfuß G. (2023). Concomitant Latent Pulmonary Vascular Disease Leads to Impaired Global Cardiac Performance in Heart Failure with Preserved Ejection Fraction. Eur. J. Heart Fail..

[20] Wolter J.S., Schulz A., Lange T., Kriechbaum S.D., Kutty S., Kowallick J.T., Treiber J.M., Rolf A., Sossalla S., Hasenfuß G. (2025). Exercise-Induced Out-Of-Proportion Increase in Afterload and Impaired Right Ventricular Contractile Reserve in HFpEF. ESC Heart Fail..

[21] Schulz A., Kuttenkeuler L., Backhaus S.J., Lange T., Otto J., Gronwald J., Evertz R., Kowallick J.T., Hasenfuß G., Schuster A. (2025). Pathophysiological and prognostic relevance of exercise CMR-derived pulmonary artery compliance in patients with suspected diastolic dysfunction and normal right ventricular function. Eur. Heart J. Imaging Methods Pract..

[22] Backhaus S.J., Schulz A., Lange T., Rösel S.F., Schmidt-Schweda L.S., Kutty S., Kowallick J.T., Treiber J., Rolf A., Sossalla S. (2025). Insights from serial cardiovascular magnetic resonance imaging show early progress in diastolic dysfunction relates to impaired right ventricular deformation. Sci. Rep..

[23] Petit T., Claessen G., Claeys M., La Gerche A., Claus P., Ghysels S., Delcroix M., Ciarka A., Droogne W., Van Cleemput J. (2021). Right Ventricular and Cyclic Guanosine Monophosphate Signalling Abnormalities in Stages B and C of Heart Failure with Preserved Ejection Fraction. ESC Heart Fail..

[24] Schulz A., Schellinger I.N., Backhaus S.J., Adler A.S., Lange T., Evertz R., Kowallick J.T., Hoffmann A., Matek C., Tsao P.S. (2024). Association of Cardiac MRI–derived Aortic Stiffness with Early Stages and Progression of Heart Failure with Preserved Ejection Fraction. Radiol. Cardiothorac. Imaging.

[25] Orizu C., Jha M., Myerson L., Dong Z.J., Neisius U., McCarthy I., Lakshminarayan D., Manning W.J., Tsao C.W. (2024). Increased Aortic Stiffness With Acute Exercise in Heart Failure: Assessment by Cardiovascular Magnetic Resonance. Am. J. Cardiol..

[26] Schulz A., Backhaus S.J., Lange T., Evertz R., Kutty S., Kowallick J.T., Hasenfuß G., Schuster A. (2024). Impact of Epicardial Adipose Tissue on Cardiac Function and Morphology in Patients with Diastolic Dysfunction. ESC Heart Fail..

[27] Burrage M.K., Hundertmark M., Valkovič L., Watson W.D., Rayner J., Sabharwal N., Ferreira V.M., Neubauer S., Miller J.J., Rider O.J. (2021). Energetic Basis for Exercise-Induced Pulmonary Congestion in Heart Failure With Preserved Ejection Fraction. Circulation.

[28] Eisman A.S., Shah R.V., Dhakal B.P., Pappagianopoulos P.P., Wooster L., Bailey C., Cunningham T.F., Hardin K.M., Baggish A.L., Ho J.E. (2018). Pulmonary Capillary Wedge Pressure Patterns During Exercise Predict Exercise Capacity and Incident Heart Failure. Circ. Heart Fail..

[29] Borlaug B.A., Kane G.C., Melenovsky V., Olson T.P. (2016). Abnormal right ventricular-pulmonary artery coupling with exercise in heart failure with preserved ejection fraction. Eur. Heart J..

[30] Backhaus S.J., Schulz A., Lange T., Evertz R., Kowallick J.T., Hasenfuß G., Schuster A. (2024). Rest and exercise-stress estimated pulmonary capillary wedge pressure using real-time free-breathing cardiovascular magnetic resonance imaging. J. Cardiovasc. Magn. Reson..

[31] Le T.-T., Bryant J.A., Ting A.E., Ho P.Y., Su B., Teo R.C.C., Gan J.S.-J., Chung Y.-C., O’Regan D.P., Cook S.A. (2016). Assessing exercise cardiac reserve using real-time cardiovascular magnetic resonance. J. Cardiovasc. Magn. Reson..

[32] Beaudry R.I., Samuel T.J., Wang J., Tucker W.J., Haykowsky M.J., Nelson M.D. (2018). Exercise cardiac magnetic resonance imaging: A feasibility study and meta-analysis. Am. J. Physiol. Regul. Intgr. Comp. Physiol..

[33] Ochs A., Nippes M., Salatzki J., Weberling L.D., Riffel J., Müller-Hennessen M., Giannitsis E., Osman N., Stehning C., André F. (2021). Dynamic Handgrip Exercise: Feasibility and Physiologic Stress Response of a Potential Needle-Free Cardiac Magnetic Resonance Stress Test. Front. Cardiovasc. Med..

[34] Bingel A., Messroghli D., Weimar A., Runte K., Salcher-Konrad M., Kelle S., Pieske B., Berger F., Kuehne T., Goubergrits L. (2022). Hemodynamic Changes During Physiological and Pharmacological Stress Testing in Patients With Heart Failure: A Systematic Review and Meta-Analysis. Front. Cardiovasc. Med..

[35] Kramer C.M., Barkhausen J., Bucciarelli-Ducci C., Flamm S.D., Kim R.J., Nagel E. (2020). Standardized cardiovascular magnetic resonance imaging (CMR) protocols: 2020 update. J. Cardiovasc. Magn. Reson..

[36] Fletcher G.F., Ades P.A., Kligfield P., Arena R., Balady G.J., Bittner V.A., Coke L.A., Fleg J.L., Forman D.E., Gerber T.C. (2013). Exercise Standards for Testing and Training: A Scientific Statement From the American Heart Association. Circulation.

[37] Ghanbari F., Schulz A., Morales M.A., Rodriguez J., Street J.A., Arcand K., Johnson S., Pierce P., Hoeger C.W., Tsao C.W. (2025). Free-breathing single-beat exercise cardiovascular magnetic resonance with generative artificial intelligence for evaluation of volumetric and functional cardiac indices: A reproducibility study. J. Cardiovasc. Magn. Reson..

[38] Bossuyt P.M., Reitsma J.B., Bruns D.E., Gatsonis C.A., Glasziou P.P., Irwig L., Lijmer J.G., Moher D., Rennie D., De Vet H.C.W. (2015). STARD 2015: An updated list of essential items for reporting diagnostic accuracy studies. Br. Med. J..

[39] Chandrasekaran P.S., Chen C., Liu Y., Arshad S.M., Crabtree C., Tong M., Han Y., Ahmad R. (2025). Accelerated real-time cine and flow under in-magnet staged exercise. J. Cardiovasc. Magn. Reson..

[40] Pednekar A., Kocaoglu M., Wang H., Tanimoto A., Tkach J.A., Lang S., Taylor M.D. (2023). Accelerated Cine Cardiac MRI Using Deep Learning-Based Reconstruction: A Systematic Evaluation. J. Magn. Reson. Imaging.

[41] Nayak K.S., Nielsen J.-F., Bernstein M.A., Markl M., Gatehouse P.D., Botnar R.M., Saloner D., Lorenz C., Wen H., Hu B.S. (2015). Cardiovascular magnetic resonance phase contrast imaging. J. Cardiovasc. Magn. Reson..

[42] Schuster A., Stahnke V.-C., Unterberg-Buchwald C., Kowallick J.T., Lamata P., Steinmetz M., Kutty S., Fasshauer M., Staab W., Sohns J.M. (2015). Cardiovascular magnetic resonance feature-tracking assessment of myocardial mechanics: Intervendor agreement and considerations regarding reproducibility. Clin. Radiol..

[43] Schulz-Menger J., Bluemke D.A., Bremerich J., Flamm S.D., Fogel M.A., Friedrich M.G., Kim R.J., Von Knobelsdorff-Brenkenhoff F., Kramer C.M., Pennell D.J. (2020). Standardized image interpretation and post-processing in cardiovascular magnetic resonance—2020 update. J. Cardiovasc. Magn. Reson..

[44] Collins G.S., Moons K.G.M., Dhiman P., Riley R.D., Beam A.L., Van Calster B., Ghassemi M., Liu X., Reitsma J.B., Van Smeden M. (2024). TRIPOD+AI statement: Updated guidance for reporting clinical prediction models that use regression or machine learning methods. Br. Med. J..

[45] Schulz A., Wallace T.E., Chow K., Bi X., Amyar A., Rodriguez J., Ghanbari F., Maron M.S., Rowin E.J., Kellmann P. (2025). Quantitative myocardial blood flow and perfusion reserve with exercise cardiovascular magnetic resonance. J. Cardiovasc. Magn. Reson..

[46] Crabtree C.D., Stoner J., Chandrasekaran P.S., Liu Y., Arshad S.M., Castillo A.M., Chen C., Tong M.S., Simonetti O.P., Ahmad R. (2026). Feasibility of real-time in-magnet exercise cardiovascular MRI with an individualized exercise protocol: Quantitative assessment at low, intermediate, and high exercise intensities. J. Cardiovasc. Magn. Reson..

[47] Messroghli D.R., Moon J.C., Ferreira V.M., Grosse-Wortmann L., He T., Kellman P., Mascherbauer J., Nezafat R., Salerno M., Schelbert E.B. (2016). Clinical recommendations for cardiovascular magnetic resonance mapping of T1, T2, T2* and extracellular volume: A consensus statement by the Society for Cardiovascular Magnetic Resonance (SCMR) endorsed by the European Association for Cardiovascular Imaging (EACVI). J. Cardiovasc. Magn. Reson..

[48] Schulz A., Mittelmeier H., Wagenhofer L., Backhaus S.J., Lange T., Evertz R., Kutty S., Kowallick J.T., Hasenfuß G., Schuster A. (2024). Assessment of the cardiac output at rest and during exercise stress using real-time cardiovascular magnetic resonance imaging in HFpEF-patients. Int. J. Cardiovasc. Imaging.

[49] Backhaus S.J., Schmermund B.N., Rieth A.J., Rademann M., Kriechbaum S.D., Wolter J.S., Wiedenroth C.B., Schulz A., Lange T., Treiber J.M. (2026). Calculation of pulmonary capillary wedge pressure including left atrial function is superior to morphology alone and accurately identifies elevated filling pressures in left heart disease. J. Cardiovasc. Magn. Reson..

[50] Ghanbari F., Rodriguez J., Morales M.A., Ngo L.H., Tsao C.W., Robbins J.M., Gopal D.M., Systrom D.M., Waxman A.B., Manning W.J. (2025). Exercise cardiovascular magnetic resonance myocardial dynamic index: A non-invasive imaging marker associated with cardiac dyspnea. J. Cardiovasc. Magn. Reson..

[51] Jain C.C., Borlaug B.A. (2020). Performance and Interpretation of Invasive Hemodynamic Exercise Testing. Chest.

[52] Craven T.P., Tsao C.W., La Gerche A., Simonetti O.P., Greenwood J.P. (2020). Exercise cardiovascular magnetic resonance: Development, current utility and future applications. J. Cardiovasc. Magn. Reson..

[53] Ghanbari F., Gopal D.M., Ngo L.H., Rodriguez J., Waxman A.B., Manning W.J., Nezafat R. (2026). Exercise cardiac magnetic resonance biventricular volumetric reserve in heart failure with preserved ejection fraction. Eur. J. Heart Fail..

[54] Oliveira-Gomes D.D., Brazile T.L., Wakeham D.J., Macnamara J.P., Hearon C.M., Zamani S.K., Nelson M.D., Levine B.D., Sarma S. (2026). Effect of Epicardial Adipose Tissue on Cardiac Function and Exercise Hemodynamics in Patients With Heart Failure With Preserved Ejection Fraction. Vis. Inf. Commun. Interact..

[55] Heidenreich P.A., Bozkurt B., Aguilar D., Allen L.A., Byun J.J., Colvin M.M., Deswal A., Drazner M.H., Dunlay S.M., Evers L.R. (2022). 2022 AHA/ACC/HFSA Guideline for the Management of Heart Failure: A Report of the American College of Cardiology/American Heart Association Joint Committee on Clinical Practice Guidelines. Circulation.

[56] Edlund J., Östenson B., Åkesson J., Heiberg E., Arheden H., Steding-Ehrenborg K. (2026). Atrioventricular area difference assessed by exercise cardiovascular magnetic resonance shows impaired diastolic filling in patients with heart failure. J. Appl. Physiol..

[57] Ghanbari F., Rodriguez J., Gopal D.M., Morales M.A., Waxman A.B., Manning W.J., Nezafat R. (2026). Exercise Cardiovascular Magnetic Resonance Work–Volume Loop Model in Heart Failure with Preserved Ejection Fraction. J. Cardiovasc. Magn. Reson..

[58] Schweitzer R., De Marvao A., Shah M., Inglese P., Kellman P., Berry A., Statton B., O’Regan D.P. (2025). Establishing Cardiac MRI Reference Ranges Stratified by Sex and Age for Cardiovascular Function during Exercise. Radiol. Cardiothorac. Imaging.

[59] Alogna A., Faragli A., Kolp C., Doeblin P., Tanacli R., Confortola G., Oetvoes J., Perna S., Stehning C., Nagel E. (2023). Blood-Oxygen-Level Dependent (BOLD) T2-Mapping Reflects Invasively Measured Central Venous Oxygen Saturation in Cardiovascular Patients. JACC Cardiovasc. Imaging.

