# Supplementary material for: Exercise Cardiovascular Magnetic Resonance for the Diagnosis and Phenotyping of Heart Failure with Preserved Ejection Fraction: A Comprehensive Review
Source: Diagnostics (Basel). 2026 Oct 23;16(21):3462. doi: 10.3390/diagnostics16213462 (PMC13761445; doi:10.3390/diagnostics16213462)
Supplement: Supplementary file 1 [file diagnostics-16-03462-s001.zip › diagnostics-4642324-supplementary.pdf]

Supplementary Table S1A. Search strategy and eligibility criteria

Item	Details
Databases searched	MEDLINE, Embase, Web of Science Core Collection, and Scopus
Search period	Database inception to 1 September 2026
Language	English
Core concepts	HFpEF or suspected HFpEF; cardiovascular magnetic resonance; dynamic exercise or exercise stress
Complementary technical concepts	Real-time CMR, feasibility, reproducibility, reference values, phase-contrast flow, feature tracking, compressed sensing, deep-learning reconstruction, spectroscopy, phosphorus magnetic resonance spectroscopy, blood oxygen level-dependent imaging, and oxygen measurements
Additional searching	Backward reference screening of eligible articles and relevant reviews; forward citation searching of key Ex-CMR studies
Inclusion criteria	Full-text human studies evaluating in-magnet exercise CMR or CMR acquired immediately after dynamic exercise; adult patients with suspected or established HFpEF; technical studies directly informing Ex-CMR acquisition, reproducibility, or interpretation; studies reporting diagnostic, phenotypic, prognostic, feasibility, or mechanistic findings relevant to Ex-CMR
Exclusion criteria	Animal or paediatric studies; case reports; editorials; narrative reviews; conference abstracts without a corresponding full publication; pharmacological stress studies without dynamic exercise; studies without a relevant CMR-based exercise assessment; studies limited to resting CMR unless directly relevant to differential diagnosis or interpretation

Data extracted	Study design; sample size; participant characteristics; HFpEF definition; invasive or non-invasive reference standard; exercise protocol; scan timing; CMR technique; analyzable scan rate and exclusions; diagnostic or prognostic estimates; follow-up outcomes; cohort overlap and external validation status
Evidence appraisal	QUADAS-2 was used for diagnostic studies with invasive exercise haemodynamics as the reference standard. Prediction models, feasibility studies, and prognostic analyses were interpreted according to their design and validation status rather than assigned a pooled quality score.

Note: The search strategy combined disease, cardiovascular magnetic resonance, exercise, and complementary technical concepts. Database-specific syntax is provided in Supplementary Table S1B. Abbreviations: 31P-MRS, phosphorus-31 magnetic resonance spectroscopy; BOLD, blood oxygen level-dependent; CMR, cardiovascular magnetic resonance; Ex-CMR, exercise cardiovascular magnetic resonance; HFpEF, heart failure with preserved ejection fraction.

Supplementary Table S1B. Database-specific search strings

Database	Search strategy
MEDLINE	(("heart failure with preserved ejection fraction"[Title/Abstract] OR HFpEF[Title/Abstract] OR "diastolic heart failure"[Title/Abstract]) AND ("Magnetic Resonance Imaging"[Mesh] OR "cardiovascular magnetic resonance"[Title/Abstract] OR "cardiac magnetic resonance"[Title/Abstract] OR "cardiac MRI"[Title/Abstract] OR "exercise CMR"[Title/Abstract] OR Ex-CMR[Title/Abstract] OR "real-time CMR"[Title/Abstract]) AND ("Exercise Test"[Mesh] OR exercis*[Title/Abstract] OR "exercise stress"[Title/Abstract] OR "exercise-induced"[Title/Abstract] OR "physical stress"[Title/Abstract] OR ergometer[Title/Abstract] OR bicycle[Title/Abstract] OR cycling[Title/Abstract]))
MEDLINE: complementary technical search	(("cardiovascular magnetic resonance"[Title/Abstract] OR "cardiac magnetic resonance"[Title/Abstract] OR "cardiac MRI"[Title/Abstract] OR "exercise CMR"[Title/Abstract] OR Ex-CMR[Title/Abstract] OR "real-time CMR"[Title/Abstract]) AND (exercis*[Title/Abstract] OR "exercise stress"[Title/Abstract] OR ergometer[Title/Abstract] OR bicycle[Title/Abstract] OR cycling[Title/Abstract]) AND (feasibility[Title/Abstract] OR reproducibility[Title/Abstract] OR repeatability[Title/Abstract] OR "reference values"[Title/Abstract] OR "normal values"[Title/Abstract] OR "healthy volunteers"[Title/Abstract] OR "phase contrast"[Title/Abstract] OR "feature tracking"[Title/Abstract] OR "compressed sensing"[Title/Abstract] OR "deep learning"[Title/Abstract] OR spectroscopy[Title/Abstract] OR "31P-MRS"[Title/Abstract] OR BOLD[Title/Abstract] OR oxygen[Title/Abstract]))
Embase	('heart failure with preserved ejection fraction':ti,ab,kw OR hfpef:ti,ab,kw OR 'diastolic heart failure':ti,ab,kw) AND ('nuclear magnetic resonance imaging'/exp OR 'cardiovascular magnetic resonance':ti,ab,kw OR 'cardiac magnetic resonance':ti,ab,kw OR 'cardiac mri':ti,ab,kw OR 'exercise cmr':ti,ab,kw OR ex-cmr:ti,ab,kw OR 'real time

	cmr':ti,ab,kw) AND ('exercise test'/exp OR exercis*:ti,ab,kw OR 'exercise stress':ti,ab,kw OR 'exercise induced':ti,ab,kw OR 'physical stress':ti,ab,kw OR ergometer:ti,ab,kw OR bicycle:ti,ab,kw OR cycling:ti,ab,kw)
Web of Science Core Collection	TS=(("heart failure with preserved ejection fraction" OR HFpEF OR "diastolic heart failure") AND ("cardiovascular magnetic resonance" OR "cardiac magnetic resonance" OR "cardiac MRI" OR "exercise CMR" OR Ex-CMR OR "real-time CMR") AND (exercise* OR "exercise stress" OR "exercise-induced" OR "physical stress" OR ergometer* OR bicycle OR cycling))
Scopus	TITLE-ABS-KEY(("heart failure with preserved ejection fraction" OR hfpef OR "diastolic heart failure") AND ("cardiovascular magnetic resonance" OR "cardiac magnetic resonance" OR "cardiac mri" OR "exercise cmr" OR ex-cmr OR "real-time cmr") AND (exercise* OR "exercise stress" OR "exercise-induced" OR "physical stress" OR ergometer* OR bicycle OR cycling))
Date restriction	Limits were applied to include records published on or before 1 September 2026. For databases permitting only annual filtering, all 2026 records were manually checked and records published after the cut-off date were excluded.

Supplementary Table S2. Structured appraisal of the primary clinical and technical evidence for exercise cardiovascular magnetic resonance in heart failure with preserved ejection fraction

Study	Design, population, and cohort relationship	Reference standard or primary endpoint	Main contribution	Key limitation and evidentiary position
Backhaus et al. HFpEF-Stress Trial	Prospective study of 75 symptomatic patients with preserved LVEF and echocardiographic features suggestive of diastolic dysfunction; 68 participants received final classification, including 34 with haemodynamically defined HFpEF and 34 with non-cardiac dyspnoea.	Resting and exercise pulmonary capillary wedge pressure measured by right-heart catheterisation.	Established real-time free-breathing Ex-CMR as a non-invasive approach to assess chamber volumes, atrial function, and dynamic cardiac responses in suspected HFpEF.	Foundational diagnostic study. It was a selected single-programme cohort; CMR and invasive testing were performed on separate days; diagnostic performance requires independent external validation.
Backhaus et al. RT-CMR report and final HFpEF-Stress Trial outcomes	Secondary reports from the HFpEF-Stress programme, based substantially on the same clinically phenotyped population.	Invasive resting and exercise haemodynamics; clinical follow-up for cardiovascular hospitalisation.	Demonstrated that exercise left atrial long-axis strain provided greater discrimination of HFpEF than resting strain.	These reports are related analyses of the original cohort, not independent replication. Prognostic analyses included relatively few events.

Backhaus et al. left atrioventricular coupling index study	Secondary analysis of 68 HFpEF-Stress participants.	Resting and exercise PCWP; cardiovascular hospitalisation during follow-up.	Exercise and resting LACI correlated with PCWP and were associated with cardiovascular hospitalisation.	LACI is a derived volumetric ratio assessed in the same cohort as the parent trial. Findings support physiological relevance but not a validated diagnostic or prognostic threshold.
Backhaus et al. estimated PCWP using real-time Ex-CMR	HFpEF-Stress programme analysis using real-time free-breathing CMR at rest and during exercise.	Invasive resting and exercise PCWP.	Left atrial volume-based estimated PCWP showed better discrimination during exercise than at rest; exercise AUC was 0.81 versus 0.73 at rest.	A multivariable model output rather than direct pressure measurement. Calibration, threshold stability, and generalisability across scanners, rhythm status, body size, and exercise protocols remain unproven.
Schulz et al. exercise cardiac output validation	Study of 68 symptomatic patients undergoing real-time phase-contrast CMR and right-heart catheterisation within 24 hours.	Cardiac output measured invasively at rest and during exercise.	Supported the feasibility of quantifying exercise cardiac output by real-time phase-contrast CMR.	CMR modestly underestimated cardiac output; examinations were not simultaneous, and different workload or physiological states may have

				contributed to disagreement. This is quantitative validation rather than HFpEF diagnostic validation.
Backhaus et al. functional CMR model for elevated filling pressure	Derivation and validation cohorts including different left-heart disease and pulmonary hypertension phenotypes.	Resting and exercise elevated filling pressure.	A model incorporating left atrial reservoir strain and indexed LV end-systolic volume identified elevated exercise filling pressure more effectively than morphology alone.	A prediction-model study in a heterogeneous population. It is not an HFpEF-specific, independently validated diagnostic rule.
Ghanbari et al. myocardial dynamic index	Exercise CMR study of 93 patients with dyspnoea; the cardiac dyspnoea group included stage B heart failure, exercise-induced HFpEF, and mildly reduced LVEF.	Clinical cardiac versus non-cardiac dyspnoea classification.	The myocardial dynamic index integrated exercise power, stroke volume index, and LV mass; AUC was 0.91 for cardiac dyspnoea.	The endpoint was broader than invasively defined HFpEF, and work rate was embedded in the index. This is exploratory composite-marker evidence rather than direct HFpEF diagnostic evidence.
Schuster et al.	Analysis of 67 HFpEF-Stress	Exercise pulmonary	Exercise right	The haemodynamic threshold

latent pulmonary vascular disease study	participants; 24 met the study-specific definition of latent pulmonary vascular disease.	vascular resistance ≥1.74 Wood units.	ventricular peak-flow index better discriminated latent pulmonary vascular disease than resting measurement.	was study-specific, and the study derived candidate markers in an overlapping cohort. It supports right ventricular–pulmonary vascular phenotyping, not stand-alone diagnosis of pulmonary vascular disease.
Morales et al. myocardial torsional reserve study	Exercise CMR tagging study; 94 participants were enrolled and 67 were analysable after exclusions for arrhythmia, slice mismatch, or triggering problems.	Comparison among exercise-induced HFpEF, overt HFpEF, non-cardiac dyspnoea, and healthy controls classified using invasive assessment.	Demonstrated reduced post-exercise torsional reserve in exercise-induced and overt HFpEF.	The substantial exclusion rate limits feasibility and generalisability. Post-exercise tagging is a mechanistic approach and currently lacks a clinically validated threshold.
Ghanbari et al. biventricular volumetric reserve study	Retrospective analysis of a prospectively recruited multicentre cohort: 40 healthy controls, 27 non-cardiac dyspnoea participants, and 73 participants across a study-defined HFpEF	Biventricular end-diastolic and end-systolic volume reserve; study-defined HFpEF stage and pulmonary hypertension status.	Identified impaired LV end-diastolic volume reserve across the HFpEF spectrum and six biventricular reserve response patterns.	Cross-sectional, post-exercise acquisition, and data-derived response patterns. The proposed phenotypes require replication and validation against outcomes or treatment

	spectrum. All Ex-CMR scans were acquired at one imaging centre.			response.
Backhaus et al. haemodynamic force study	Case-control substudy from the HFpEF-Stress programme.	Resting cine-derived intraventricular haemodynamic forces and late-diastolic filling measures.	Identified lower longitudinal haemodynamic forces, systolic peaks, and impulses in HFpEF.	Primarily resting CMR and mechanistic analysis; it does not establish exercise-specific diagnostic performance.
Schulz et al. sex-specific functional reserve study	Sex-stratified analysis of the HFpEF-Stress population.	Sex-related differences in exercise ventricular and atrial functional reserve.	Identified lower exercise RV stroke volume and adverse atrial functional responses in women with HFpEF.	Small subgroup analysis from an overlapping cohort. Findings are hypothesis-generating and should not define sex-specific thresholds.
Backhaus et al. left atrial roof enlargement study	Cross-sectional three-dimensional left atrial shape analysis in the HFpEF-Stress programme.	Resting versus exercise-induced elevation of filling pressure.	Identified local left atrial roof enlargement in exercise-induced HFpEF and more diffuse enlargement in overt HFpEF.	Secondary morphological analysis in the parent cohort. It suggests a potential imaging phenotype but lacks independent validation.
Schulz et al.	Analysis of 68 HFpEF-Stress	Associations of	Higher epicardial	Cross-sectional association

epicardial adipose tissue study	participants stratified by epicardial adipose tissue burden.	epicardial adipose tissue with HFpEF classification, native T1, and exercise strain responses.	adipose tissue burden was associated with more overt HFpEF and impaired atrial and ventricular strain reserve.	study. Causality cannot be inferred, and findings may vary with adipose-tissue compartment, obesity severity, and exercise posture.
Wolter et al. right ventricular contractile reserve study	Exercise haemodynamic and CMR analysis in HFpEF.	Relationship between exercise afterload and right ventricular contractile reserve.	Showed an out-of-proportion rise in afterload with inadequate right ventricular contractile compensation during exercise.	Mechanistic study from the same specialist research programme; it supports physiological phenotyping rather than a diagnostic threshold.
Schulz et al. pulmonary artery compliance study	Study of 63 participants with suspected diastolic dysfunction and normal resting RV ejection fraction.	Exercise pulmonary artery area pulsatility and stroke-volume-normalised capacitance; cardiovascular events during follow-up.	Exercise pulmonary artery compliance measures were reduced in HFpEF and associated with later cardiovascular events.	Imaging surrogates do not directly measure pulmonary artery pressure or resistance. Prognostic estimates were based on few events and had wide confidence intervals.
Backhaus et al. serial right	Serial CMR follow-up of HFpEF-Stress participants without	Longitudinal change in resting and exercise RV	Linked progression of diastolic dysfunction	Small longitudinal study from an overlapping

ventricular deformation study	interatrial shunt implantation.	long-axis strain.	with worsening RV deformation.	programme; strain is load-dependent and does not isolate intrinsic RV contractility.
Petit et al. right ventricular–pulmonary vascular and sildenafil study	Mechanistic study of 16 HFpEF patients, 7 patients with concentric remodelling and impaired relaxation, and 8 healthy controls.	Exercise CMR, simultaneous central haemodynamics, cyclic guanosine monophosphate measurements, and response to a single 50 mg sildenafil dose.	Demonstrated impaired exercise RV–pulmonary vascular reserve in HFpEF-spectrum disease.	Small acute intervention study. The neutral response to one sildenafil dose cannot be extrapolated to long-term PDE5 inhibition or other HFpEF therapies.
Schulz et al. aortic pulse-wave velocity study	Analysis of 48 HFpEF-Stress participants with non-cardiac dyspnoea, occult HFpEF, or overt HFpEF.	Resting aortic pulse-wave velocity and its relationship with PCWP, strain, and natriuretic peptides.	Resting aortic stiffness was higher in occult and overt HFpEF than in non-cardiac dyspnoea.	Resting vascular measurement rather than direct exercise assessment; small overlapping cohort.
Orizu et al. recovery-phase aortic distensibility study	Twenty-four heart failure patients and 26 controls; only 10 participants had HFpEF.	Aortic distensibility before and approximately 1 minute after submaximal supine	Demonstrated recovery-phase aortic stiffening in the overall heart failure group.	Very small HFpEF subgroup and recovery rather than peak-exercise imaging; no significant HFpEF-specific

		cycling.		difference was shown.
Burrage et al. energetics and pulmonary congestion study	Multimodal CMR study of 11 healthy controls, 9 participants with type 2 diabetes, 14 with HFpEF, and 9 with cardiac amyloidosis.	Resting myocardial PCr/ATP, 20-W exercise chamber reserve, and exercise-induced lung proton-density signal.	Lower PCr/ATP was associated with impaired cardiac reserve and increased exercise-related lung-water signal.	Small cross-sectional study with resting spectroscopy and low-level exercise. It identifies mechanistic associations, not a clinically actionable metabolic phenotype.
Schulz et al. quantitative exercise perfusion study	Prospective cohort of 108 recruited participants; 89 had analyzable paired rest and post-exercise perfusion data, including 34 HFpEF patients.	Quantitative myocardial blood flow and perfusion reserve acquired 45–60 seconds after exercise.	Demonstrated feasibility of recovery-phase quantitative perfusion CMR and showed a major influence of achieved peak heart rate on flow measures.	Recovery-phase acquisition does not capture peak physiology. The study did not establish an HFpEF-specific perfusion threshold.
Ghanbari et al. work–volume loop study	Exercise CMR analysis of 120 participants using rest/post-exercise ventricular volumes and maximal exercise power.	Model-derived work–volume loop geometry, compliance reserve, and power-adjusted contractile reserve.	Suggested distinct volumetric and workload-adjusted responses across exercise-induced and stage C HFpEF.	Loop area is a model-derived geometric metric, not directly measured stroke work. The study requires external validation and outcome linkage.

Ghanbari et al. single-beat Ex-CMR reproducibility study	Technical study of 141 participants; technically successful scanning in 136 participants, with repeat imaging in 15 healthy individuals.	Scan success, observer reproducibility, and test–retest volumetric and functional measurements.	Supported feasibility and reproducibility of free-breathing single-beat Ex-CMR with generative artificial intelligence reconstruction.	Predominantly technical evidence; healthy-participant retesting was limited and does not establish reproducibility in elderly, obese, arrhythmic, or advanced HFpEF populations.
Schweitzer et al. exercise CMR reference-range study	Exercise CMR study in 161 healthy participants with sex- and age-stratified reference ranges.	Exercise-related ventricular volume and functional responses in healthy individuals.	Provides an initial framework for interpreting normal exercise CMR responses by sex and age.	Reference values require validation across scanners, ethnic groups, exercise protocols, and clinical populations before use as HFpEF thresholds.
Alogna et al. blood oxygen level-dependent CMR proof-of-concept study	Methodological study of oxygen-sensitive CMR measurements.	Central venous oxygen saturation estimated using blood oxygen level-dependent CMR.	Illustrates the potential of CMR to assess oxygen-related physiological signals beyond chamber function and flow.	Not an HFpEF diagnostic study and not yet an exercise-HFpEF clinical tool. It is included only as an emerging future direction.

Abbreviations: AUC, area under the receiver operating characteristic curve; CMR, cardiovascular magnetic resonance; cGMP, cyclic guanosine

monophosphate; Ex-CMR, exercise cardiovascular magnetic resonance; HFpEF, heart failure with preserved ejection fraction; LACI, left atrioventricular coupling index; LVEF, left ventricular ejection fraction; LV, left ventricular; PCr/ATP, phosphocreatine-to-adenosine triphosphate ratio; PCWP, pulmonary capillary wedge pressure; PDE5, phosphodiesterase type 5; RV, right ventricular.
